# The Traditional Japanese Medicine Rikkunshito Promotes Gastric Emptying via the Antagonistic Action of the 5-HT_3_ Receptor Pathway in Rats

**DOI:** 10.1093/ecam/nep173

**Published:** 2011-02-13

**Authors:** K. Tominaga, T. Kido, M. Ochi, C. Sadakane, A. Mase, H. Okazaki, H. Yamagami, T. Tanigawa, K. Watanabe, T. Watanabe, Y. Fujiwara, N. Oshitani, T. Arakawa

**Affiliations:** ^1^Department of Gastroenterology, Osaka City University Graduate School of Medicine, Osaka 545-8585, Japan; ^2^Tsumura Research Laboratories, Tsumura & Company, Ibaraki, Japan

## Abstract

The traditional Japanese medicine rikkunshito ameliorates the nitric oxide-associated delay in gastric emptying. Whether rikkunshito affects gastric motility associated with 5-hydroxytryptamine (serotonin: 5-HT) receptors or dopamine receptors is unknown. We examined the effects of rikkunshito on the delay in gastric emptying induced by 5-HT or dopamine using the phenol red method in male Wistar rats. 5-HT (0.01–1.0 mg kg^−1^, i.p.) dose dependently delayed gastric emptying, similar to the effect of the 5-HT_3_ receptor agonist 1-(3-chlorophenyl) biguanide (0.01–1.0 mg kg^−1^, i.p.). Dopamine also dose dependently delayed gastric emptying. The 5-HT_3_ receptor antagonist ondansetron (0.04–4.0 mg kg^−1^) and rikkunshito (125–500 mg kg^−1^) significantly suppressed the delay in gastric emptying caused by 5-HT or 1-(3-chlorophenyl) biguanide. Hesperidin (the most active ingredient in rikkunshito) suppressed the 5-HT-induced delayed gastric emptying in a dose-dependent manner, the maximum effect of which was similar to that of ondansetron (0.4 mg kg^−1^). The improvement obtained by rikkunshito or ondansetron in delaying gastric emptying was completely blocked by pretreatment with atropine. Rikkunshito appears to improve delay in gastric emptying via the antagonistic action of the 5-HT_3_ receptor pathway.

## 1. Introduction

The traditional Japanese medicine rikkunshito is used in Japan to treat various disorders of the gastrointestinal (GI) tract [1–3]. Diseases associated with GI tract disorders cause a range of symptoms such as abdominal fullness, bloating, nausea, vomiting and postprandial early satiety, the physiological mechanisms of which are critically involved in GI motility disorders. It is hypothesized that rikkunshito may have an effect on GI motility. In a previous study by our research group, rikkunshito was shown to ameliorate the delay in gastric emptying induced by N^G^-nitro-l-arginine (an inhibitor of nitric oxide (NO) synthase), and that its main active ingredient for improving motility disorders of the stomach could be hesperidin, identified from its methanol fraction by highly porous polymer chromatography [4]. Gastrointestinal motility is regulated not only by NO but also various neurotransmitters such as 5-hydroxytryptamine (5-HT: serotonin), dopamine, catecholamines, histamine and acetylcholine. Investigating if rikkunshito can affect the GI motility associated with 5-HT or dopamine pathways is crucial because various prokinetic agents affecting 5-HT or dopamine receptors are often used for diseases associated with GI motility disorders.

In the present study, we examined if rikkunshito and its main active ingredient hesperidin could ameliorate the delay in gastric emptying induced by 5-HT or dopamine.

## 2. Methods

### 2.1. Drugs and Chemicals

Rikkunshinto has eight constituents: *Glycyrrhizae* radix (4.7%), *Zingiberis* rhizoma (2.3%), *Atractylodis lanceae* rhizoma (18.6%), *Zizyphi* fructus (9.3%), *Aurantii nobilis* pericarpium (9.3%), *Ginseng* radix (18.6%), *Pinelliae* tuber (18.6%) and *Hoelen* (18.6%). Rikkunshito was obtained from Tsumura and Company (Tokyo, Japan) as a dried powder extract. Rikkunshito was extracted in hot water from a mixture of these eight constituents. Rikkunshito used in this study was standardized by the following three procedures: (i) identification of spots derived from the respective crude drugs by thin-layer chromatography; (ii) evaluation of the amount of absolute ethanol-soluble components: conform to item of “Diethyl ether-soluble extract" in the “Extract content" in the “Crude Drug Test" in the General Tests, Methods and Apparatus described in the Japanese Pharmacopoeia; and (iii) quantification of the contents of glycyrrhizic acid and hesperidin by liquid chromatography (no specified value has been published by Tsumura & Co.). A voucher specimen (number 2020043010) has been deposited in Tsumura and Company. Ondansetron hydrochloride (Zofran injection) was purchased from GlaxoSmithKline (Middlesex, UK). Mosapride citrate was kindly donated by Dainippon Sumitomo Pharmaceutical Company (Osaka, Japan). 5-HT 5-hydroxytryptamine hydrochloride: serotonin, atropine sulfate salt, 1-(3-Chlorophenyl) biguanide hydrochloride, phenol red and Tween-80 were purchased from Sigma-Aldrich (St. Louis, MO, USA). Hesperidin was obtained from Tsumura and Company. In all *in vivo* experiments, rikkunshito was dissolved in distilled water and administered (p.o.) in 10 ml kg^−1^ into rats. Ondansetron and atropine sulfate salt were dissolved in physiological (0.9%) saline and injected (s.c,) in 2 ml kg^−1^ into rats. 5-HT, dopamine and 1-(3-chlorophenyl) biguanide were dissolved in physiological saline and injected (i.p.) in 2 ml kg^−1^ into rats.

### 2.2. Animals

Animal experiments were carried out in accordance with the institutional guidelines of Tsumura and Company after obtaining permission from the Laboratory Animal Committee and approval from the Animal Ethics Committee of Osaka City University. Experiments were designed to minimize the number of animals used and their suffering.

Male Wistar rats (age 9 weeks) were obtained from Japan SLC (Hamamatsu, Japan). Rats were allowed to acclimatize to their surroundings for ≥1 week. They had free access to water and standard laboratory food. They were maintained in a facility at a temperature of 25 ± 1°C, relative humidity of 55 ± 25%, and controlled lighting with lights on from 07:00 to 19:00 h daily.

### 2.3. Measurement of Gastric Emptying in Rats

The experiment was conducted in accordance with the method reported in our previous articles [4, 5]. Briefly, rats were fasted for 24 h, and then 1 ml of phenol red (0.1 mg ml^−1^) as a nonabsorbable marker was injected into the stomach of each rat. Each rat was killed 15 min after administration of phenol red (except those killed immediately after injection to recover the entire dose of phenol red) and the stomach immediately removed. The stomach was cut into several pieces in 10 ml of Na_2_HPO_4_ solution (0.1  mol l^−1^) to collect the gastric contents, including phenol red (S_1_ solution). One millilitre of S_1_ solution was collected to determine the phenol red concentration of S_1_ solution, and the residual S_1_ solution added to 1 ml of phenol red solution at 0.1 mg ml^−1^ to prepare S_2_ solution. The absorbance values of S_1_ and S_2_ solutions were measured as optical density (OD)_1_ and OD_2_ at 560 nm using a UV-1200 spectrophotometer (Shimadzu Company, Kyoto, Japan). The rate of gastric emptying was calculated as follows:

(1) $$\text{Amount of phenol red in stomach }\left(\mu \text{g}\right)=\left(\frac{100-{\text{S}}_{1}}{{\text{S}}_{2}-{\text{S}}_{1}}\right)\times {\text{S}}_{1}$$

Note: S_1_≤ OD_1_>* xa* + *b*, S_2_≤ OD_2_>* xa* + *b*

〈*a*〉 and 〈*b*〉 are coefficients obtained from the standard curve for phenol red.

(2) $$\text{Gastric emptying rate}\;\left(\%\right)=100-\left(\frac{A}{B}\right)\times 100$$

*A*: amount of phenol red remaining in each stomach (*μ*g) and *B*: amount of phenol red recovered from the stomach immediately after administration of phenol red (*μ*g).

### 2.4. Changes in Gastric Emptying by Various Drugs (5-HT, 5-HT_3_ Agonist, 5-HT_4_ Agonists and Dopamine) in Rats

The 5-HT and the 5-HT_3_ agonist 1-(3-chlorophenyl) biguanide at 0.01–1.0 mg kg^−1^ were injected (i.p.) before administration of phenol red. The 5-HT_4_ agonist mosapride citrate at 0.3–3.0 mg kg^−1^ was administered (p.o.) 60 min before administration of phenol red. Dopamine (0.4 mg kg^−1^, i.p.) was injected immediately before administration of phenol red according to previous report [6]. Each vehicle was administered to control rats instead of 5-HT, 5-HT_3_ agonist, 5-HT_4_ agonist or dopamine using the same schedule. Gastric emptying was evaluated using the method described above.

### 2.5. Therapeutic Efficacy for the Delay in Gastric Emptying Induced by 5-HT, Dopamine and a 5-HT_3_ Agonist [1-(3-Chlorophenyl) biguanide] by Rikkunshito or Ondansetron in Rats

According to previous reports [6], 5-HT (0.02 mg kg^−1^, i.p.) or dopamine (0.4 mg kg^−1^, i.p.) was injected immediately, and the 5-HT_3_ agonist 1-(3-chlorophenyl) biguanide (0.4 mg kg^−1^, i.p.) was injected 30 min before administration of phenol red. Rikkunshito (125, 250 or 500 mg kg^−1^, p.o.) was administered 120 min before injection of 5-HT or dopamine, and 90 min before injection of 1-(3-chlorophenyl) biguanide. The human dose of rikkunshito is 4 g (converted to extract)/day, as described in a book, “MAN-BYO-KAI-SHUN" (Wan bing hui chun). For a human adult weighing 60 kg, the dose is 4000 mg/60 kg = 66.6 mg kg^−1^; a 10× higher dose in rats becomes 666.6 mg kg^−1^. Accordingly, the upper limit dose was set at 500 mg kg^−1^ in this study. Ondansetron (0.04, 0.4 or 4.0 mg kg^−1^, s.c.) was injected 30 min before injection of 5-HT or dopamine, and immediately before injection of 1-(3-chlorophenyl) biguanide. Distilled water was administered or vehicle was injected to control rats instead of rikkunshito or ondansetron under the same schedule. Gastric emptying was evaluated using the method described above.

### 2.6. Improvement of the 5-HT-Induced Delay in Gastric Emptying by Hesperidin in Rats

Hesperidin (0.3, 1.0 or 3.0 mg kg^−1^, p.o.) was administered 120 min before injection of 5-HT (0.02 mg kg^−1^, i.p.). Distilled water was injected into control rats instead of hesperidin under the same schedule.

### 2.7. Pretreatment with Atropine and Inhibition of the Improvement of the 5-HT-Induced Delay in Gastric Emptying by Rikkunshito or Ondansetron

5-HT (0.02 mg kg^−1^) was injected immediately before administration of phenol red. In the case of atropine treatment, atropine (1 mg kg^−1^) was injected 120 min before injection of 5-HT. Rikkunshito (500 mg kg^−1^, p.o.) was administered 120 min before injection of 5-HT, and ondansetron (0.4 mg kg^−1^, s.c.) injected 30 min before injection of 5-HT into rats.

### 2.8. Statistical Analysis

Values are expressed as mean ± SEM. The significance of differences between groups was evaluated using Student's *t*-test or one-way analysis of variance (ANOVA) followed by Dunnett's multiple comparison test. *P* < .05 were considered significant.

## 3. Results

### 3.1. Amelioration of the 5-HT- and Dopamine-Induced Delay in Gastric Emptying by Rikkunshito in Rats

**Figure 1:**
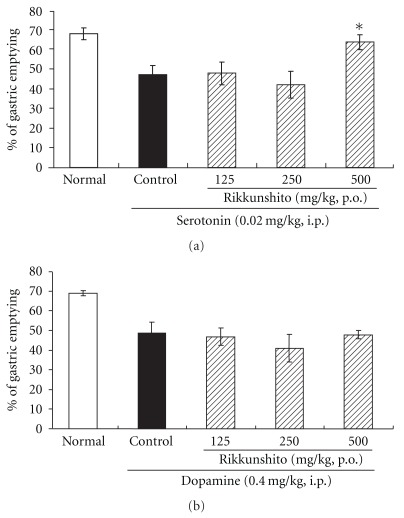
Amelioration of the 5-HT- (a) and dopamine-induced (b) delay in gastric emptying by rikkunshito in rats: (a) rikkunshito administration at a high dose of 500 mg kg^−1^ ameliorated the 5-HT (0.02 mg kg^−1^)-induced delay in gastric emptying, but rikkunshito at a lower dose (125 or 250 mg kg^−1^) did not; (b) rikkunshito at any of the doses used in this study did not reverse the delay caused by dopamine (0.4 mg kg^−1^); each column represents the mean ± SEM. of eight rats. *P < .01: significantly different from the control group.

We initially reconfirmed the delay in gastric emptying induced by injection of 5-HT (0.02 mg kg^−1^, i.p.) and dopamine (0.4 mg kg^−1^, i.p.), which was consistent with previous reports [6]. Rikkunshito administration at a high dose of 500 mg kg^−1^ ameliorated the 5-HT-induced delay in gastric emptying, but rikkunshito at a lower dose (125 or 250 mg kg^−1^) did not (Figure 1(a)). Rikkunshito at any of the doses used did not reverse the delay caused by dopamine (Figure 1(b)).

### 3.2. Changes in Gastric Emptying by 5-HT, 5-HT_3_ [1-(3-Chlorophenyl) biguanide] and 5-HT_4_ (Mosapride) Agonists in Rats

**Figure 2:**
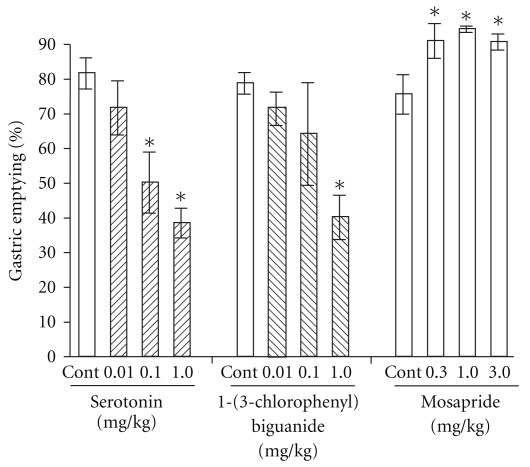
Changes in gastric emptying by 5-HT, 5-HT_3_, 1-(3-chlorophenyl) biguanide, and 5-HT_4_ (mosapride) agonists in rats: intraperitoneal injection of 5-HT (0.1 and 1.0 mg kg^−1^) significantly delayed gastric emptying compared with the controls (from 81.7 ± 4.5 to 50.2 ± 8.8 and 38.5 ± 4.3%) in a dose-dependent manner. The 5-HT_3_ agonist 1-(3-chlorophenyl) biguanide significantly delayed gastric emptying, similar to 5-HT (from 78.8 ± 3.1 to 40.2 ± 6.4%), whereas the 5-HT_4_ agonist mosapride (0.3–3.0 mg kg^−1^) accelerated it (from 75.6 ± 5.7 to 90.7 ± 2.3%). Each column represents the mean ± SEM. of eight rats. *P < .01: significantly different from the control group.

Intraperitoneal injection of 5-HT significantly delayed gastric emptying compared with controls in a dose-dependent manner (controls, 81.7 ± 4.5%; at 0.1 and 1.0 mg kg^−1^, 50.2 ± 8.8 and 38.5 ± 4.3%). The 5-HT_3_ agonist, 1-(3-Chlorophenyl) biguanide also significantly delayed gastric emptying in a dose-dependent manner similar to the effects of 5-HT (from 78.8 ± 3.1 to 40.2 ± 6.4%), whereas the 5-HT_4_ agonist mosapride at 0.3–3.0 mg kg^−1^ accelerated it from 75.6 ± 5.7 to 90.7 ± 2.3% (Figure 2).

### 3.3. Amelioration of the 5-HT- and Dopamine-Induced Delay in Gastric Emptying by 5-HT Receptor Antagonists (Ondansetron) in Rats

**Figure 3:**
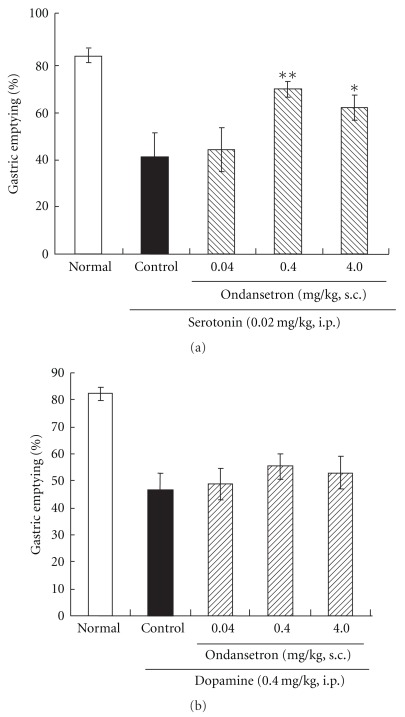
Amelioration of the 5-HT- (a) and dopamine-induced (b) delay in gastric emptying by ondansetron in rats: (A) administration of ondansetron (0.4 or 4.0 mg kg^−1^) ameliorated the 5-HT (0.02 mg kg^−1^)-induced delay in gastric emptying; (B) ondansetron at any of the doses used in this study did not reverse the delay caused by dopamine (0.4 mg kg^−1^). Each column represents the mean ± SEM of eight rats. **P < .01, *P < .05: significantly different from the control group.

The 5-HT_3_ receptor antagonist ondansetron at 0.4 and 4.0 mg kg^−1^ significantly ameliorated the 5-HT-induced delay in gastric emptying (Figure 3(a)), but it did not affect the delay induced by dopamine (Figure 3(b)). These findings suggest that intraperitoneal injection of 5-HT acts on the type-3 receptor of 5-HT.

### 3.4. Improvement of the 5-HT Agonist [1-(3-Chlorophenyl) biguanide]-Induced Delay in Gastric Emptying by Rikkunshito and Ondansetron

**Figure 4:**
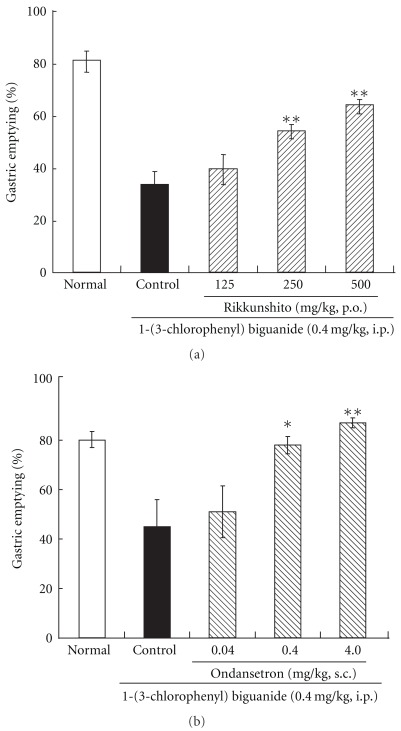
Improvement in the 5-HT_3_ agonist [1-(3-chlorophenyl) biguanide]-induced delay in gastric emptying by rikkunshito (a) and ondansetron (b): the delay induced by intraperitoneal injection of the 5-HT_3_ agonist 1-(3-Chlorophenyl) biguanide at 0.4 mg kg^−1^ (from *∼*80 to 35–40%) was suppressed by rikkunshito administration in a dose-dependent manner (a). Like rikkunshito, pretreatment with ondansetron significantly ameliorated delay of gastric emptying in a dose-dependent manner (b). Each column represents the mean ± SEM of eight rats. **P < .01, *P < .05: significantly different from the control group.

To clarify the pharmacological effect of rikkunshito on the 5-HT_3_ receptor, we examined the effects of rikkunshito or ondansetron (reference arm) on the 5-HT_3_ agonist-induced delay in gastric emptying. The delay induced by intraperitoneal injection of the 5-HT_3_ agonist 1-(3-chlorophenyl) biguanide at 0.4 mg kg^−1^ (from *∼*80% to 35–40%) was suppressed by rikkunshito administration in a dose-dependent manner (Figure 4(a)). Like rikkunshito, pretreatment with ondansetron significantly ameliorated delay of gastric emptying in a dose-dependent manner (Figure 4(b)).

### 3.5. Improvement of the 5-HT-Induced Delay in Gastric Emptying by Hesperidin

**Figure 5:**
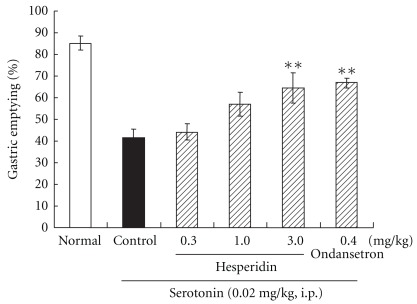
Improvement in the 5-HT-induced delay in gastric emptying by hesperidin: administration of hesperidin (one of the ingredients extracted from rikkunshito) improved the 5-HT-induced delay in gastric emptying in a dose-dependent manner. Hesperidin at a high dose (3.0 mg kg^−1^) showed a similar effect to that of ondansetron (0.4 mg kg^−1^) for this delay, but hesperidin at a lower dose (0.3 or 1.0 mg kg^−1^) did not. Each column represents the mean ± SEM of eight rats. **P < .05: significantly different from the control group.

Administration of hesperidin (one of ingredients extracted from rikkunshito), which ameliorated the delay in gastric emptying by a NO synthase inhibitor in the previous report, suppressed the 5-HT-induced delay in gastric emptying in a dose-dependent manner (Figure 5). High-dose hesperidin (3.0 mg kg^−1^) showed a similar effect to that of ondansetron (0.4 mg kg^−1^) for this delay.

### 3.6. Inhibition of the Improvement of the 5-HT-Induced Delay in Gastric Emptying by Rikkunshito or Ondansetron by Pretreatment with Atropine

**Figure 6:**
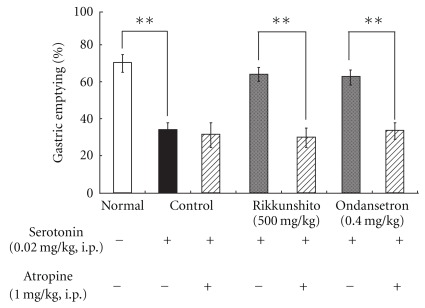
Inhibition of the improvement in 5-HT-induced delay in gastric emptying by rikkunshito and ondansetron by pretreatment with atropine: there was no effect of pretreatment with atropine alone at 1 mg kg^−1^ on 5-HT-induced delay in gastric emptying. The improvement in 5-HT-induced delay in gastric emptying by rikkunshito and ondansetron was completely blocked by pretreatment with atropine. Each column represents the mean ± SEM of eight rats. **P < .01: significantly different from each paired group.

There was no effect of pretreatment with atropine alone at 1 mg kg^−1^ on 5-HT-induced delay in gastric emptying. The improvement on 5-HT-induced delay in gastric emptying by rikkunshito or ondansetron was completely blocked by pretreatment with atropine (Figure 6).

## 4. Discussion

In the present study, we demonstrated that rikkunshito and hesperidin (the most active ingredient in rikkunshito) could improve the 5-HT-induced delay in gastric emptying in rats, the mechanism of which appears to involve the antagonistic action of the 5-HT_3_ receptor pathway.

Recently, integrative and complementary studies demonstrated various additional effects such as anti-inflammatory and anti-tumor effects of present therapeutic medicines and natural substances via targets for particular molecules [7, 8]. Those studies have critical significances in gastroenterology, and we reported the antibacterial effect of the Japanese herbal medicine, gosyuyu (wu-chu-yu) against *Helicobacter pylori*, *in vitro* and *in vivo* [9, 10]. Several studies show that Japanese herbal medicines including rikkunshito have a clinical efficacy for functional dyspepsia (FD) via improvement of GI motility disorders [11, 12]. However, there are a few evidences which demonstrated the pharmacological function of rikkunshito like this, although, recently, a part of pharmacological mechanism(s) and active ingredients of rikkunshito have been elucidated [4, 13].

Gastric motility-like emptying and accommodation is regulated at various levels such as NO, 5-HT and its receptors. Many clinically useful drugs affect gastric motility via NO or 5-HT type-3 or type-4 receptors. It has also been reported that rikkunshito has a prokinetic action mediated by NO [4, 13, 14]. If rikkunshito has another pharmacological action than that described above, it may prove that rikkunshito has an alternative significance in gastric motility similar to the combined efficacy of several drugs. In our previous study, rikkunshito could ameliorate the delay in gastric emptying induced by an inhibitor of NO synthase: N^G^-nitro-l-arginine [4]. Initially, the mechanism was thought to be mediated via NO production alone because rikkunshito includes l-arginine, a substrate of NO, in its water fraction. This hypothesis appears to be supported by previous contributions that showed that rikkunshito promotes NO-associated gastric adaptive relaxation in isolated guinea-pig stomach [13, 14]. Analysis for fractional extracts of rikkunshito revealed that there was a more potent ingredient (hesperidin) in the methanol fraction than the crude water fraction or l-arginine alone, which promotes gastric motility [4]. It was reported that hesperidin is not a substrate of NO because of a non-member of the guanidine group [15] and that it decreases NO production [16]. It was therefore demonstrated that rikkunshito and hesperidin had more potential to promote gastric motility mediated via a mechanism other than the NO-associated mechanism. These findings allow us to elucidate the effect of rikkunshito on gastric motility disorders associated with 5-HT and dopamine because these neurotransmitters (as well as NO) have a significant role in gastric motility and various prokinetic agents affecting 5-HT receptors or dopamine receptors which are often used to treat GI motility disorders.

**Figure 7:**
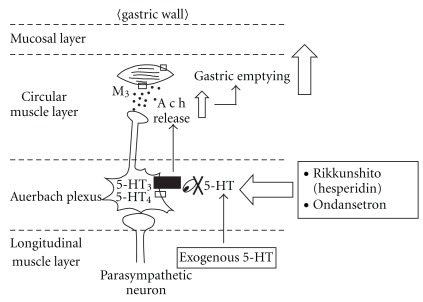
A scheme of improvement of the 5-HT-induced delay in gastric emptying by rikkunshito.

**Figure 8:**
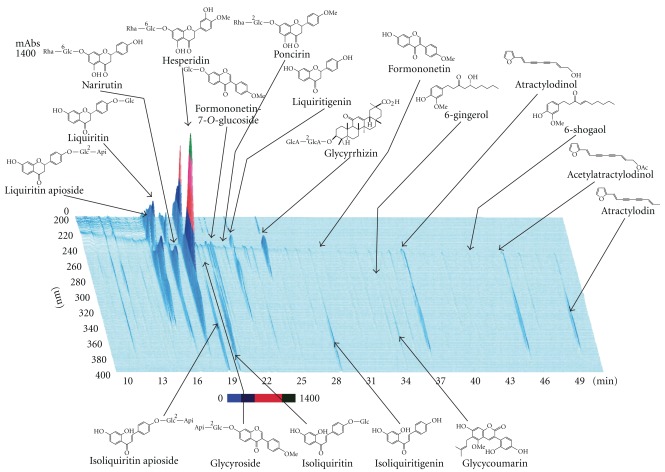
Three dimensional analysis for rikkunshito by high performance liquid chromatography.

Intraperitoneally injected 5-HT fails to cross the blood-brain barrier [17]. It is therefore likely that 5-HT binds to any 5-HT receptors located on vagal afferent fibers and enteric nerves, resulting in a delay of gastric emptying [18]. Of the seven types of 5-HT receptors previously identified, the 5-HT_3_ and 5-HT_4_ receptors participate in the regulation of sensory and motor functions of the GI tract [19, 20]. Injection of the 5-HT_3_ agonist 1-(3-chlorophenyl) biguanide delayed gastric emptying, whereas the 5-HT_4_ agonist mosapride accelerated gastric emptying in rats. Rikkunshito, hesperidin and the 5-HT_3_ receptor antagonist ondansetron ameliorated the 5-HT-induced delay in gastric emptying, whereas the 5-HT_4_ receptor antagonist SB204070 had no effect (data not shown). Rikkunshito could not affect the delay induced by dopamine. This suggests that the effect of rikkunshito on gastric motility was not non-specific. The present efficacy of rikkunshito in enhancing gastric motility may be mediated by other substances, including catecholamines, histamine, acetylcholine and ghrelin. Rikkunshito may be associated with ghrelin in the promotion of gastric motility because it can elevate the low levels of plasma ghrelin induced by cisplatin [21]. It was also reported that the dose of 5-HT used in the present study could not change the peripheral secretion and gastric production of ghrelin [21]. The pharmacological action of rikkunshito in promoting gastric motility indicated in the present study may therefore not be associated with ghrelin. It was reported that the 13 crude components contained in rikkunshito had no binding activity with the 5-HT_3_ receptor [21]. Pretreatment with atropine completely blocked the rikkunshito- and ondansetron-induced improvements in gastric emptying in the 5-HT-induced delay model described above. The 5-HT_3_ antagonistic effect on increased gastric motility is mediated by acetylcholine release, which leads to contraction of smooth muscle. Therefore, it appears that rikkunshito (like ondansetron) has 5-HT_3_ antagonist effects on gastric motility. Taken together, these findings suggest that rikkunshito and hesperidin may ameliorate the delay in gastric emptying in rats via the antagonistic action of the 5-HT_3_ receptor pathway, but not 5-HT_4_ receptor (Figure 7). Moreover, the efficacy of rikkunshito and hesperidin may appear to be a partial effect compared to that of ondansetron in this model, because their effects on the gastric emptying seemed to be weaker than that of ondansetron. However, there were no statistical significances between the normal and rikkunshito (500 mg kg^−1^) groups, and hesperidin (3.0 mg kg^−1^) showed a similar recovery in gastric emptying to ondansetron (0.4 mg kg^−1^). Thus, these slight differences among rikkunshito, hesperidin and ondansetron are thought to be derived from the differences in selectivity for the 5-HT_3_ receptor pathway. It cannot be concluded with certainty that rikkunshito has a pharmacological effect on any site of the 5-HT_3_ receptor pathway because the signal transduction pathway after binding of the specific agonist of the 5-HT_3_ receptor or direct effects of the agents on various molecules associated with this signal have not been elucidated [22, 23]. A major limitation of the present study was that the signal transduction pathway or targeted molecules after binding of the specific agonist of the 5-HT_3_ receptor has not been elucidated. We therefore could not pursue the detailed target molecules for rikkunshito through the 5-HT_3_ receptor pathway. On the other hand, it is known that other active ingredients for gastric motility are included in rikkunshito, such as 6-gingesulfonic acid, shogasulfonic acid A and atractylodin [24, 25]. However, rikkunshito consists of over a 100 compounds, and proportion of several active components of rikkunshito is small. Therefore, it is difficult for an appropriate isolation to examine each pharmacological function *in vivo*. Thus, we could not check the effect of all compounds on gastric motility. The main purpose of this study was to investigate whether rikkunshito affects gastric motility via the 5-HT pathway. Therefore, we have examined the effects of rikkunshito and hesperidin, the most active components of rikkunshito (Figure 8).

In general, it has been shown that prokinetic drugs are useful for patients with FD in a meta-analysis for prokinetic drugs using mainly cisapride [26, 27]. Prokinetic drugs are not established in the therapeutic strategy for FD patients. The reason may be that the currently available prokinetic drugs cannot comprehensively regulate gastric motility, including gastric accommodation and emptying. Different from other prokinetic drugs that have a single pharmacological action, rikkunshito has at least a possibility to accelerate gastric accommodation and emptying mediated by the NO and 5-HT_3_ receptor pathway. Rikkunshito could therefore be an alternative candidate drug for prokinetic drugs such as itopride and mosapride; this hypothesis is supported by previous clinical articles [28–30]. In general, a herbal medicine like rikkunshito has not been a firstly chosen candidate for various diseases, since it is consisted of multiple components whose pharmacological function and active ingredients have not been elucidated in detail. Therefore, rikkunshito has been recognized to be only a complementary and alternative drug. However, some recent basic researches and established evidences about rikkunshito might demonstrate not only an impact as a complementary and alternative drug but also new clinical benefits as a standardized drug.

In conclusion, we demonstrated that rikkunshito and hesperidin (a potent ingredient of rikkunshito), showed pharmacological efficacy in enhancing gastric motility via antagonism of the 5-HT_3_ receptor-associated pathway. Rikkunshito may thus prove useful in improving abdominal symptoms caused by motility dysfunction in human patients.

## 5. Funding

Grant-in-Aid for Scientific Research from the Ministry of Education, Culture, Sports, Science and Technology of Japan.
